# Association between Dietary Patterns and Metabolic Syndrome Risk Factors: A Cross-Sectional Study of Christian Orthodox Church Fasters and Non-Fasters in Greece

**DOI:** 10.3390/foods12183488

**Published:** 2023-09-19

**Authors:** Anna Kokkinopoulou, Niki Katsiki, Ioannis Pagkalos, Nikolaos E. Rodopaios, Alexandra-Aikaterini Koulouri, Eleni Vasara, Sousana K. Papadopoulou, Petros Skepastianos, Maria Hassapidou, Anthony G. Kafatos

**Affiliations:** 1 Department of Preventive Medicine and Nutrition Unit, School of Medicine, University of Crete, 71003 Crete, Greece; 2 Department of Nutritional Sciences and Dietetics, International Hellenic University, 57400 Thessaloniki, Greece; 3 School of Medicine, European University Cyprus, 2404 Nicosia, Cyprus; 4 Laboratory of Animal Physiology, Department of Zoology, School of Biology, Aristotle University of Thessaloniki, 54124 Thessaloniki, Greece; 5 Department of Medical Laboratory Studies, International Hellenic University, 57400 Thessaloniki, Greece

**Keywords:** dietary patterns, Mediterranean diet, Western diet, plant-based diet, Christian Orthodox Church fasting, metabolic syndrome

## Abstract

It is well known that the Mediterranean diet contributes to healthy living, prevention of non-communicable diseases, and longevity. A cross-sectional study was conducted with participants from Greece who follow the Mediterranean diet and were further divided into two categories: (i) Christian Orthodox Church (COC) religious fasters and (ii) non-fasters. Four-hundred individuals underwent anthropometric measurements, whereas information regarding dietary intake was collected via three 24 h diet recalls and a monthly food frequency questionnaire. Principal component analysis was performed to derive dietary patterns, whereas associations between dietary patterns and metabolic syndrome (MetS) risk factors were investigated with the general linear model. Non-fasters (*n* = 200) were found to consume significantly more beef, chicken, turkey, sausage, broth, fried potatoes, ketchup, and mustard, while consuming less seafood, snails, soya, tarama salads, fresh fruits, margarine, olives, and decaf coffee. Two distinct dietary patterns were identified in fasters: (i) the “Mixed Diet” and (ii) the “Plant-based/Fasting Diet” pattern. Moreover, three patterns were identified in non-fasters, and were identified as follows: (i) the “Western Diet”, (ii) the “Mixed Diet”, and (iii) the “Mediterranean-like Diet” pattern. No significant association was observed between dietary patterns and the prevalence of MetS in our population. Further epidemiological studies should evaluate the links between dietary patterns and MetS prevalence within the adult Greek population.

## 1. Introduction

Food represents a fundamental aspect of human life, providing nourishment and energy, but it also plays significant social and cultural role [1]. Dietary patterns refer to the composition, combination, and variety of foods and beverages consumed by individuals or populations over time [2]. During recent decades, scientific interest has focused on how dietary patterns, rather than individual nutrients and/or food items, may affect the risk of chronic diseases [3,4].

The most commonly recognized dietary patterns that have been linked to longevity and protection against non-communicable diseases (NCDs) are the Mediterranean, Nordic, and Okinawan dietary patterns [5,6,7,8,9]. Evidence of the benefits of the Mediterranean diet were first reported in the Seven Countries Studies (SCS) in the late 1950s, showing that a population of men from Crete who followed a traditional lifestyle way of living, with an (i) increased level of physical activity, (ii) consumption of traditional foods, and who (iii) fasted according to the Christian Orthodox Church (COC) recommendations, had a higher life expectancy [10,11,12]. Indeed, the COC fasting regime is part of the Mediterranean diet in Greece, which is followed for 180–200 days spread throughout the year in four major fasting periods (i.e., Christmas, Easter, Assumption, and Holy Apostoles). During these fasting periods, abstinence from red and white meat, dairy products, and eggs is mandatory, while the consumption of fish, seafood, and snails is allowed only during specific days [13].

It is shown that the extensively studied Mediterranean diet has been proven to play a key role in both the prevention and treatment of NCDs, including cardiovascular diseases, type 2 diabetes, cancer, and metabolic syndrome (MetS) [10,11,12,13,14,15,16,17,18]. MetS represents a major public health problem, being associated with the development of several cardiometabolic diseases, including type 2 diabetes and its complications, cardiovascular diseases, and non-alcoholic fatty liver disease [14,15,16,17]. MetS is diagnosed when three or more of the following joint criteria are met: (i) elevated waist circumference (according to race-specific cut off points); (ii) elevated fasting glucose levels (≥100 mg/dL); (iii) reduced high-density lipoprotein (HDL) cholesterol [<40 mg/dL (1.0 mmol/L) in males, and <50 mg/dL (1.3 mmol/L) in females]; (iv) increased triglyceride (TG) levels (≥150 mg/dL, 1.7 mmol/L); and (v) elevated blood pressure (BP) levels (systolic BP ≥ 130 and/or diastolic BP ≥ 85 mm Hg) [18]. According to the literature, following a Western type of diet has been linked with increased incidence of MetS, while a Mediterranean diet has been in favor of reducing all independent risk factors of MetS [19,20,21,22]. Also, plant-based diets [23,24,25] and vegetarian dietary patterns [26,27] have been linked with positive outcomes of reducing all related MetS risk factors.

As MetS remains a public health issue, the relationship of the Mediterranean diet, and more specifically, the COC recommendations along with its risk factors, needs further investigation. Up to date, a few studies focusing on the effects of COC on MetS risk factors have been carried out in Greece [28,29,30,31,32], Africa [33,34,35], and the USA [36,37]. The initial aim of this study was to identify the dietary patterns followed by a COC fasting population and a non-fasting population in Greece, and secondly, to investigate any relationships between the dietary patterns and MetS risk factors and prevalence.

## 2. Methods

### 2.1. Study Population

This cross-sectional study was conducted in Thessaloniki, Greece. An email invitation for this study was sent to two local Universities, i.e., the Alexander Technological Educational Institute of Thessaloniki and the Aristotle University of Thessaloniki, as well as to local churches. An initial diet questionnaire and a list with inclusion criteria were used to determine eligible participants. Fasters were defined as those following the COC fasting for the last 12 consecutive years or since childhood, whereas those who did not follow any restrictive diet were identified as non-fasters. Inclusion criteria also included the following: (i) age > 18 years and (ii) being in good general health, whereas exclusion criteria included the following: (i) being unable to provide written or informed consent, (ii) being pregnant or breastfeeding, and (iii) having long-term co-morbidities (e.g., diabetes mellitus). This study’s protocol was approved by the Bioethics Committee of the Alexander Technological Educational Institute of Thessaloniki (31-5/5679).

All individuals were informed about this study’s protocol and had the opportunity to ask questions to researchers before providing their consent. A total of 54 individuals from the initial 454 individuals that expressed interest in participation were excluded due to exclusion criteria. Finally, the remaining 400 individuals were included in this study, consisting of 200 fasters and 200 non-fasters. More details regarding this study’s design were already published [28].

### 2.2. Anthropometric, Biochemical and Dietary Data

A validated questionnaire was used to collect anthropometric and biochemical data, medical history, and dietary intake [38]. A trained nutritionist performed all anthropometric measurements and supervised the completion of the questionnaires. All participants were asked to fast prior to the scheduled appointment for at least 10 h and to abstain from physical activity the previous day. In terms of biochemical measurements, blood samples were collected from all participants and analyses were performed in a certified lab. In terms of anthropometric measurements, participants’ (i) weight was measured with a digital scale to the nearest 100 g (TANITA UM075, Amsterdam, The Netherlands); (ii) height was measured with a portable stadiometer to the nearest 0.5 cm (TANITA HR001, Leicester, UK); (iii) waist and hip circumference were measured with a body girth tape to the nearest 0.5 cm (SECA 201, Hamburg, Germany); (iv) body composition and, more specifically, body fat, muscle mass, and body water were measured with a bioimpedance analyzer (BODYSTAT 1500, Warwickshire, UK) following the bioelectrical impedance analysis (BIA) method; and (v) blood pressure (BP) was measured with an electronic BP monitor to the nearest 0.5 mmHg (Omron, Hoffman Estates, IL, USA). Data on clinical history, education, marital status, physical activity levels, sedentary time spending watching television and/or using the computer, time sleeping, smoking status, and alcohol consumption, among others, were collected via open-ended and closed questions. More details regarding data collection and measurements were already published [28,29].

A combination of methods was used for recording dietary intake, including three 24 h diet recalls [39], one food frequency questionnaire (FFQ) [40], and a nutritional behavior questionnaire [38], as shown in the validated questionnaire of the NutriHeAl study that was conducted on a Greek populace. All questionnaires were answered with the help of a nutritionist; food replicas and household measures were used for the accuracy of portion sizes, and the Food Processor v11.7 nutrition analysis software (ESHA, Salem, OR, USA) was used to analyze food records. For each food item, participants were asked to choose one of the following consumption options: (i) never/rarely, (ii) 1–3 times/month, (iii) 1–2 times/week, (iv) 3–6 times/week, (v) 1 time/day, and (vi) >2 times/day.

### 2.3. Statistics

The SPSS v.21 statistical package (SPSS, Chicago, IL, USA) was used to perform the analyses. One of the most common methods to identify dietary patterns is principal component analysis (PCA) [4,41]. Scree plots and interpretability of each component were used to determine the appropriate number of components that described the dietary patterns. Foods positively and negatively associated with each dietary pattern were derived with linear combination [41,42]. Chi-squared test, one-way analysis of variance (ANOVA), general linear model, and logistic regression analyses were also performed to investigate the associations between dietary patterns, anthropometric and biochemical data, as well as MetS risk factors and prevalence. A two-sided *p*-value of 0.05 was set for statistical significance.

## 3. Results

Among the 400 participants, 200 were fasters (131 women, mean age 43.4 ± 16.7 years) and 200 were non-fasters (126 women, mean age 41.9 ± 17.3 years). Diastolic BP was significantly lower in fasters (*p* = 0.003), whereas significantly more non-fasters were smokers (*p* < 0.001), had more weekly workouts during their free time (*p* = 0.021), slept more (*p* = 0.008), and spent more time in front of a computer/mobile screen (*p* = 0.043) and watching TV (*p* < 0.001). Detailed results can be seen in the Supplementary Materials Table S1 and the comparisons were already published previously [28].

Fasters had significantly lower consumption of beef (*p* = 0.007), chicken (*p* = 0.001), turkey (*p* = 0.003), pork sausage (*p* = 0.003), broth (*p* = 0.012), fried potatoes (*p* = 0.001), ketchup (*p* = 0.002), and mustard (0.014). With regard to beef consumption, it was noticed that 16.5% of fasters never consumed and 6% of non-fasters did; chicken was never consumed by 15% of fasters vs. 5% of non-fasters; turkey was never consumed by 85.5% of fasters vs. 79% of non-fasters; pork sausages were never consumed by 54% of fasters vs. 44% of non-fasters; broth was never consumed by 63% of fasters vs. 53% of non-fasters; fried potatoes were never consumed by 36% of fasters vs. 17.5% of non-fasters; ketchup was never consumed by 59.5% of fasters vs. 49% of non-fasters; and mustard was never consumed by 39.5% of fasters vs. 31.5% of non-fasters. Furthermore, fasters had significantly higher consumption of seafood (*p* = 0.008), snails (*p* = 0.026), soya (*p* = 0.001), tarama salad (*p* = 0.041), fresh fruits (*p* = 0.022), margarine (*p* = 0.024), olives (*p* = 0.006), and decaf coffee (*p* = 0.023). In more details, seafood was consumed more than two times per week by 57.5% of fasters vs. 37% of non-fasters; snails were consumed one to six times per week by 4.5% of fasters vs. 1% of non-fasters; soya was consumed by one to six times per week by 10% of fasters vs. 1.5% of non-fasters; tarama salad was consumed by one to six times per week by 17.5% of fasters vs. 9.5% of non-fasters; fresh fruits were consumed once and/or more than two times per day by 60% of fasters vs. 52% of non-fasters; olives were consumed once and/or more times per day by 19% of fasters vs. 10% of non-fasters; margarine was consumed one to six times per week by 23.5% of fasters vs. 16% of non-fasters; and decaf coffee was consumed once and/or more than two times per day by 5% of fasters vs. 1.5% of non-fasters.

One-hundred and fifty-three foods and beverages were initially included for the analysis. Only those consumed by ≥5% of the population were grouped into 61 interpretable and meaningful categories for the PCA. For example, food items from FFQ ‘lemonade/orangeade with sugar’, ‘fizzy drink cola with sugar’, and ‘fizzy drink with sugar other’ were all grouped in a new category/food group labeled ‘soft drinks with sugar’. Food groups with the corresponding food item(s) are presented in Supplementary Table S2. PCA revealed three distinct dietary patterns in the whole population of this study, i.e., the “Western Diet”, the “Mediterranean-like Diet”, and the “Plant-based/Fasting Diet” pattern, which explained 17.5% of the variance when combined. The names of the identified dietary patterns were selected for descriptive purposes and were based on the highest coefficient scores (≥0.3). The “Western Diet” pattern included 18 food groups and explained 7.2% of the variance. The “Mediterranean-like Diet” pattern was positively associated with 14 food groups and explained 5.6% of the variance. Lastly, the “Plant-based/Fasting Diet” pattern was related to 11 food groups and explained 4.7% of the variance. A factor loading of at least |0.3| was needed to characterize the positive and negative association with the specific dietary pattern. Supplementary files provide details of the constituent foods comprising the 61 food groups analyzed using PCA (Supplementary Table S2), as well as the factor loadings for the population of this study (Supplementary Table S3).

In the group of fasters, two distinct dietary patterns explained 14% of the variance, i.e., 7.9% the “Mixed Diet” pattern and 6.1% the “Plant-based/Fasting Diet” pattern, respectively. The first one, the “Mixed Diet” pattern, encompassed full-fat milk and yogurt, processed meat, white meat, bread, potatoes, pasta, rice, and other white cereals, biscuits and cakes, savory snacks, pizza, and energy drinks, among others. The “Plant-based/Fasting Diet” pattern was characterized by a high consumption of wholegrain bread, fruits, vegetables (all categories, including raw, not raw, and cooked dishes), low-fat milk, yogurt, and cheese, olive oil, seafood, nuts, and traditional confectionary (including halvah and spoon sweets) foods. Results from the PCA with food groups and factor loadings are shown in Table 1.

In the group of non-fasters, three dietary patterns were derived with PCA, explaining 18.7% of the variance, i.e., 7.6% the “Western Diet”, 5.9% the “Mixed Diet” pattern, and 5.1% the “Mediterranean-like Diet” pattern, respectively. The “Western Diet” pattern was characterized by a high consumption of full-fat milk and yogurt, salty baked, savory snacks, biscuits, ice cream, white and other bread, potatoes, fruit juices, traditional foods such as moussaka and pastitsio, pizza, soft drinks, chocolate confectionery, and white pasta, rice, and other cereals, among others. The “Mixed Diet” pattern involved the consumption of low-fat milk, yogurt and cheese, wholegrain breakfast cereals, wholegrain bread, liquor, beer, wine, beer, traditional spirits (including ouzo, tsipouro, and raki), soft drinks without sugar, canned fish, and rusks, among others. The last pattern, the “Mediterranean-like Diet”, was characterized by a high intake of olive oil, tahini, nuts, full-fat yogurt and cheese, fruits, vegetables (not raw), wholegrain bread, eggs, fresh fish, and coffee, among others. Table 2 shows the full details of the food groups and factor loadings for these dietary patterns.

Independent associations between socio-economic and lifestyle characteristics, MetS risk factors, and all identified dietary patterns were examined with multiple linear regression models. In Table 3, it is observed that fasters closer to the “Mixed Diet” pattern during a non-fasting period spend more hours being physical active (*p* = 0.039), had elevated waist circumference (*p* = 0.05), and elevated BP levels (*p* = 0.002), while no other significant association was observed between the rest of the variables. On the contrary, fasters closer to the “Plant-based/Fasting Diet” pattern during a non-fasting period had no significant associations with the selected variables.

Table 4 presents findings for non-fasters. In more details, those closer to the “Western Diet” pattern were women (*p* = 0.039), those closer to the “Mixed Diet” pattern had no observed association, while those who were closer to a “Mediterranean-like Diet” pattern were married (*p* = 0.001) and had increased waist circumference (*p* = 0.024). No significant association was observed between lifestyle habits in both groups (fasters and non-fasters), such as smoking, alcohol consumption, and dietary intake. Importantly, in both study groups, no relationship was observed between distinct dietary patterns and MetS prevalence.

We also conducted a gender analysis. In men (*n* = 143), two distinct dietary patterns explained 15.2% of the variance. The first one, the “Western Diet” pattern, explained 8.7% of the variance, and included a high consumption of soft drinks with sugar, other bread, butter, savory snacks, salty baked products, salad dressing dips, full-fat milk, soft drinks without sugar, cacao and chocolate powder, puddings, fruit juices, biscuits and cakes, other oils, processed meat, white bread and products, traditional confectionery, beer, liquor, energy drinks, pastitsio, other potatoes, pasta, rice, and other white cereals, and other breakfast cereals. The “Mediterranean-like Diet” pattern explained 6.4% of the variance, and was characterized by increased consumption of fruits, wholegrain grain bread and products, olive oil and olives, low-fat yogurt, rusks, nuts, vegetables raw, wholegrain breakfast cereals, cereal bar, low-fat milk, fresh fish, low-fat cheese and vegetables (not raw). Results from the PCA with food groups and factor loadings are shown in Supplementary Table S4. In women (*n* = 257), the same two dietary patterns were derived and explained 13.2% of the variance (7.5% and 5.7%, respectively). The first one, the “Western Diet” pattern, was characterized by a high consumption of salad dressing dips, ice cream, fruit juices, other bread, puddings, full-fat milk, pastitsio, pizza, savory snacks, soft drinks with sugar, legumes, potatoes (other), traditional confectionary, processed meat, cacao and chocolate powder, biscuits and cakes, other oils, and salty baked products. The “Mixed Diet” pattern involved a high consumption of low-fat milk, low-fat yogurt, liquor, cereal bar, wholegrain breakfast cereals, wine, traditional spirits, other breakfast cereals, beer, low-fat cheese, coffee, soft drinks without sugar, wholegrain bread and products, and rusks. PCA results are presented in Supplementary Table S5.

A further analysis was carried out based on gender and diet status combined. For male fasters, we identified the “Mixed Diet” pattern explaining 10.1% of the variance and the “Plant-based/Fasting Diet” pattern explaining 7.9% of the variance. The “Mixed Diet” pattern was positively associated with the consumption of fruit juices, soft drinks with sugar, other bread, butter, other oils, cacao and chocolate powder drinks, full-fat milk, processed meat, pastitsio and moussaka, biscuits and cakes, pasta, rice, and other white cereals, full-fat yogurt, potatoes (other), savory snacks, fish canned, salad dressing dips, puddings, energy drinks, full-fat cheese, other breakfast cereals, salty baked products, white bread and other products, red meat, tea, sugar, honey, and marmalade. The “Plant-based/Fasting Diet” pattern was characterized by a high consumption of fruits, wholegrain bread and other products, vegetables (raw), cereal bar, low-fat cheese, low-fat milk, nuts, low-fat yogurt, olive oil and olives, wholegrain breakfast cereals, vegetables, homemade dishes, traditional confectionery, savory snacks, rusks, soya products, other seafood, vegetables (not raw), and pasta, rice, and other wholegrain cereals.

For female fasters, we identified the “Western Diet” pattern explained 7.7% of the variance and the “Mixed Diet” pattern explained 6.3% of the variance. The “Western Diet” pattern was characterized by a high consumption of salad dressing dips, other bread, processed meat, pastitsio and moussaka, puddings, white meat, fruit juices, energy drinks, pizza, savory snacks, ice cream, potatoes (other), other breakfast cereals, full-fat milk, legumes, soft drinks with sugar, biscuits and cakes, full-fat yogurt, nougat and halva, chocolate confectionery, and pasta, rice, and other white cereals. The “Mixed Diet” pattern was positively associated with the consumption of wholegrain bread and products, coffee, traditional spirits, vegetables (raw), cereal bar, low-fat milk, olive oil and olives, low-fat yogurt, wholegrain breakfast cereals, rusks, nougat and halvah, fruits, other fat spreads, sugar, honey, marmalade, tea, nuts, vegetables, and homemade dishes.

In the group of non-fasters, we identified the same two dietary patterns in both men and women: the “Western Diet” and the “Mixed Diet” patterns. These patterns explained 16.3% and 14.8% of the variance in men and women, respectively. In male non-fasters, the “Western Diet” pattern explained 9.5% of the variance, and was characterized by a high consumption of other bread, soft drinks with sugar, savory snacks, soft drinks without sugar, salty baked products, beer, puddings, salad dressing dips, full-fat milk, traditional confectionary, butter, chocolate confectionary, biscuits and cakes, liquor, other seafood, cacao and chocolate powder, white bread and other white grains, whole grain breakfast cereals, cereal bars, energy drinks and low-fat cheese. The “Mixed Diet” pattern explained 6.8% of the variance and was positively associated with the consumption of wholegrain bread and other white grains, olive oil and olives, fruits, wine, rusks, low-fat milk, pasta, rice, and other wholegrain cereals, eggs, nuts, vegetables, homemade dishes, low-fat yogurt, fresh fish, beer, and wholegrain breakfast cereals.

In non-fasters women, the “Western Diet” pattern explained 8.3% of the variance and was characterized by a high consumption of salad dressing dips, ice cream, puddings, fruit juices, full-fat milk, soft drinks with sugar, cacao and chocolate powder drinks, other bread products, pizza, salty baked products, legumes, savory snacks, pastitsio and moussaka, traditional confectionary, potatoes (other), cereal bars, other breakfast cereals, other oils, white bread and products, biscuits, and cakes. The “Mixed Diet” pattern, explaining 6.5% of the variance, was associated with a high consumption of low-fat milk, liquor, low-fat cheese, other breakfast cereals, beer, low-fat yogurt, cereal bar, soft drinks without sugar, wine, and wholegrain breakfast cereals.

## 4. Discussion

In this cross-sectional study, fasters were found to consume significantly less beef, chicken, turkey, sausages, broth, fried potatoes, ketchup, and mustard, while significantly more seafood, snails, soya, tarama salad, fresh fruits, margarine, olives, and decaf coffee, during a non-fasting period. Our findings answer previously raised questions regarding the exact differences in the dietary intake of COC fasters during non-fasting periods [28,43]. This is of great importance as results reveal that fasters adopted the COC fasting recommendations not only during fasting periods but also followed a healthier dietary pattern throughout the year. It is known that fasting is associated with healthy dietary habits during fasting periods. For example, 99 COC fasters in the USA were reported to consume significantly more legumes, soya products, nuts, seeds, wholegrain products, and fresh fruits, as well as significantly less red meat, poultry, processed meat, dairy products, and eggs during the 48-day Easter fasting period [36]. Similarly, fasters who were members of two different Greek monasteries (*n* = 10) were reported to consume significantly more legumes, fish, shellfish, snails, and nuts during the Easter fasting period when compared with a non-fasting period [44]. In agreement with our results, in another Greek COC fasting population (*n* = 38), it was found that fasters consumed significantly more legumes, potatoes, fruits, and vegetables after Christmas, Lent, and the Assumption fasting periods when compared with non-fasters (*n* = 29) [45,46]. These findings support the hypothesis that fasters tend to adopt healthy dietary habits even in non-fasting periods.

In this study, two distinct dietary patterns were identified in fasters, i.e., the “Mixed Diet” and the “Plant-based/Fasting Diet” pattern, while three dietary patterns were identified in non-fasters, i.e., the “Western Diet”, the “Mixed Diet” and the “Mediterranean-like Diet” pattern. Similarly, in the ATTICA study (*n* = 3.042 Greek healthy adults), three dietary patterns were identified as follows: the “healthy dietary choices”, the “between”, and the “unhealthy dietary choices” pattern [47]. The “healthy dietary choices” pattern, characterized by fish, vegetables, fruits, cereals, legumes, and potatoes, was linked with a lower incidence of cardiovascular disease [47]. All these foods positively correlated to the “Plant-based/Fasting Diet” pattern that was identified in fasters in this study. In the Vyronas study in Greece with 2118 participants, two dietary patterns were derived, i.e., the “Junk food” and the “Vegetarian/healthy” pattern, followed by individuals who also adopted a healthier lifestyle, such as an increased level of physical activity and absence from smoking [48]. In another study with 146 metabolically unhealthy obese Greek adults, four distinct dietary patterns were extracted, i.e., the “Western-type”, the “Mediterranean-like diet”, the “healthy”, and the “animal meat and sauces” pattern [32]. Elevated body weight, body fat, waist circumference, and BMI were positively correlated with the “Western-type” and the “animal meat and sauces” dietary patterns, whereas increased HDL levels and high physical activity scores were positively associated with the “Mediterranean-like diet” in another study with 146 Greek adults [49]. Furthermore, in the MAST4HEALTH study, an increased adherence was found in the “Western” and the “Plant-based” patterns among Greek participants, similar to our study [50]. Notably, in a secondary analysis of data collected form the ATTICA study, it was shown that Greeks were gradually deviating from the traditional Mediterranean diet pattern towards a Western-type dietary pattern [51].

Among 16,008 middle-aged Spanish adults in the SUN study, a “Mediterranean Diet” pattern, characterized by higher consumption of vegetables, fish, seafood, fruits, and olive oil, was linked to reduced mortality [52], whereas the “Western Diet” pattern, including an increased consumption of ultra-processed foods, was positively associated with an increased risk for all-cause mortality [53]. In the NutriNet-Santé study with a sample of 105,832 individuals, three major dietary patterns were identified, i.e., the “Healthy”, the “Traditional”, and the “Western” pattern [35], similar to the three dietary patterns that were derived in our sample (*n* = 400). The incidence of inflammatory bowel diseases was positively associated with the “Healthy” pattern and negatively related to the “Western” pattern [54].

Gender analysis revealed different dietary patterns in men and women, i.e., the “Western Diet” and the “Mediterranean-like Diet” pattern in men, and the “Western Diet” and the “Mixed Diet” pattern in women. Similarly, in a Swedish adult population (*n* = 952 women and 788 men), three distinct dietary patterns were identified in women (i.e., the “Healthy”, the “Swedish traditional”, and the “Light-meal” pattern), whereas two patterns were identified in men (i.e., the “Healthy” and the “Swedish traditional” pattern) [36]. In contrast, among 479 adult Indonesians, four dietary patterns were derived in both sexes, i.e., the “Noodle, oil, and salty sea products”; the “Meat, vegetable, oil, and fruit”; the “Vegetable, non-oil, and milk”; and the “Staples, Oil, and Sweet” dietary pattern [37]. Similarly, in a Japanese adult population (6080 women and 3723 men), the same five dietary patterns were identified in both genders, i.e., the “healthy”, the “Western”, the “seafood”, the “bread”, and the “dessert” dietary pattern [38]. Increased BMI was positively associated with the “Western”, the “seafood”, and the “dessert” dietary pattern, and negatively associated with the “healthy” and the “bread” dietary pattern [55].

With regard to the links between dietary patterns and MetS, the Korean National Health and Nutrition Examination Survey involving data from 9069 participants (3777 men and 5292 women) found that a high carbohydrate intake was strongly associated with a higher risk of MetS in women [21]. In another study among 276 Polish adults, three distinct dietary patterns were identified, i.e., the Western, Prudent, and Low Food diet pattern; rare consumption of fish was a predictor of more severe MetS cases [22]. Furthermore, among 11,305 Korean adults aged 40 years or older, three similar, but not identical, dietary patterns were empirically identified in men and women [56]. In sum, MetS was inversely associated with the “vegetables/seaweeds” pattern in men, but positively related in women, whereas the association between the “meat/poultry/seafood” pattern and MetS was U-shaped in men and inversed in women. The “non-traditional/non-staple foods” pattern was negatively related to MetS in both genders [56].

This study has certain limitations. For example, all participants were volunteers and their categorization into the two study groups was based on their self-report of adhering to the COC fasting recommendations since their childhood or at least the last 12 consecutive years. Furthermore, the sample size is not nationally representative; hence, the results cannot be generalized. However, our study population was one of the largest in relation to COC fasters [13,57]. Furthermore, the dietary patterns were based on the self-reporting FFQ and 24 h recalls; hence, the supervision of the participants from a trained nutritionist was essential. Overall, this study could represent the basis for further research in this field, evaluating the associations of dietary patterns with MetS components and the prevalence among fasters. There is an urgent need to support sustainable food systems; thus, research interest has focused on interventions for sustainable healthy diets during recent years, such as the Mediterranean Diet [58,59,60,61], while religious fasting, and more specifically COC fasting, is regarded as an important asset to be used in public health nutrition strategies [62].

## 5. Conclusions

This cross-sectional study found that fasters followed the COC fasting recommendations during a non-fasting period, contributing to a healthier and more sustainable way of life. Furthermore, distinct dietary patterns were identified in fasters and non-fasters, as well as in men and women. No association was found between the identified dietary patterns and MetS prevalence. Further research is needed to thoroughly investigate the food items characterizing each dietary pattern and their level of processing in order to contribute to public health strategies.

## Figures and Tables

**Table 1:** Score coefficients from principal component analysis regarding food groups consumed by fasters (*n* = 200).

Food Group(s) *	Mixed Diet Pattern	Plant-Based/Fasting Diet Pattern
	Variance Explained 7.9%	Variance Explained 6.1%
Beer	0.180	0.090
Biscuits and cakes	0.388	−0.070
Butter	0.291	−0.023
Cacao and chocolate powder	0.318	0.020
Cereal bar	0.095	0.468
Cheese (full fat)	0.177	−0.112
Cheese (low fat)	0.035	0.412
Chocolate confectionery	0.224	−0.019
Coffee	−0.132	0.421
Eggs	0.151	−0.041
Energy drinks	0.354	−0.121
Fish (canned)	0.246	0.218
Fresh fish	0.077	−0.029
Fruit juices	0.510	−0.066
Fruits	−0.165	0.528
Ice cream	0.283	0.104
Legumes	0.184	0.070
Liquor	0.170	0.173
Milk (full fat)	0.347	−0.219
Milk (low fat)	0.111	0.422
Nougat halva	0.323	0.316
Nuts	−0.055	0.417
Olive oil olives	−0.211	0.397
Other bread	0.563	0.066
Other breakfast cereals	0.352	0.150
Other fat spreads	0.234	0.060
Other oils	0.288	0.034
Other seafood	0.055	0.328
Pasta, rice, and other white cereals	0.311	0.014
Pasta, rice, and other wholegrain cereals	0.295	0.087
Pastitsio	0.469	−0.088
Pies	0.160	−0.085
Pizza	0.386	0.140
Potatoes (boiled)	0.079	0.118
Potatoes (other)	0.395	−0.073
Processed meat	0.514	−0.040
Puddings	0.480	0.043
Red meat	0.281	−0.018
Rusks	0.057	0.415
Salad dressings dips	0.554	0.182
Salty baked products	0.277	0.084
Savory snacks	0.399	0.103
Soft drinks with sugar	0.462	−0.250
Soft drinks without sugar	0.266	0.113
Soya products	0.194	0.102
Sugar honey marmalade	0.269	0.113
Tahini and spreads	−0.186	0.186
Tea	0.310	0.296
Traditional confectionery	0.234	0.296
Traditional spirits	0.067	0.323
Vegetables homemade dishes	0.043	0.363
Vegetables (not raw)	−0.263	0.352
Vegetables (raw)	−0.109	0.451
Water	−0.199	0.244
White bread and products	0.129	0.130
White meat	0.340	−0.111
Wholegrain breakfast cereals	0.119	0.401
Wholegrain bread and products	−0.090	0.575
Wine	0.096	0.149
Yogurt (full fat)	0.337	0.080
Yogurt (low fat)	−0.008	0.420

* Foods with the highest factor loadings ≥0.3 and those between 0.25 to 0.3 are positively related to a dietary pattern. Foods with low factor loadings, and therefore less likely to be part of a dietary pattern, are between −0.25 and −0.3 and ≤−0.3.

**Table 2:** Food groups and respective factor loadings in non-fasters (*n* = 200).

Food Group(s) *	Western Diet Pattern	Mixed Diet Pattern	Mediterranean-like Diet Pattern
	Variance Explained 7.6%	Variance Explained 5.9%	Variance Explained 5.1%
Beer	0.108	0.407	−0.180
Biscuits and cakes	0.405	−0.186	0.131
Butter	0.317	0.16	0.274
Cacao and chocolate powder	0.463	0.109	0.130
Cereal bar	0.324	0.370	−0.065
Cheese (full fat)	0.214	−0.064	0.445
Cheese (low fat)	0.092	0.431	−0.118
Chocolate confectionery	0.298	−0.087	0.132
Coffee	−0.163	0.239	0.251
Eggs	0.013	0.000	0.366
Energy drinks	0.193	0.186	−0.010
Fish (canned)	−0.106	0.372	0.005
Fresh fish	−0.001	0.106	0.281
Fruit juices	0.376	0.132	−0.159
Fruits	−0.048	−0.007	0.451
Ice cream	0.433	−0.056	−0.226
Legumes	0.256	−0.062	−0.150
Liquor	0.041	0.473	−0.072
Milk (full fat)	0.545	−0.038	0.005
Milk (low fat)	0.081	0.558	−0.154
Nougat halva	0.211	0.148	0.269
Nuts	0.119	0.088	0.419
Olive oil olives	−0.078	0.137	0.537
Other bread	0.512	0.249	0.067
Other breakfast cereals	0.241	0.382	−0.115
Other fat spreads	0.135	−0.312	0.052
Other oils	0.376	−0.275	−0.003
Other seafood	0.284	0.175	0.054
Pasta, rice, and other white cereals	0.273	0.079	0.127
Pasta, rice, and other wholegrain cereals	0.111	0.213	0.096
Pastitsio	0.362	0.020	0.030
Pies	−0.022	−0.085	0.360
Pizza	0.330	0.047	−0.056
Potatoes (boiled)	0.089	0.253	−0.059
Potatoes (other)	0.333	−0.096	−0.111
Processed meat	0.208	0.138	−0.321
Puddings	0.488	0.218	−0.154
Red meat	0.045	0.056	−0.049
Rusks	0.086	0.336	0.153
Salad dressings dips	0.529	0.158	−0.361
Salty baked products	0.435	0.203	−0.089
Savory snacks	0.419	0.218	−0.34
Soft drinks with sugar	0.465	0.106	−0.238
Soft drinks without sugar	0.244	0.399	−0.269
Soya products	0.101	−0.202	−0.224
Sugar honey marmalade	0.318	−0.190	0.139
Tahini and spreads	0.087	−0.162	0.447
Tea	0.045	−0.001	0.19
Traditional confectionery	0.497	−0.112	0.181
Traditional spirits	−0.121	0.326	0.200
Vegetables homemade dishes	0.283	−0.014	0.197
Vegetables (not raw)	−0.076	−0.128	0.346
Vegetables (raw)	−0.001	0.268	0.014
Water	0.004	0.090	0.024
White bread and products	0.387	−0.003	0.093
White meat	0.144	0.215	−0.120
Wholegrain breakfast cereals	0.172	0.533	0.223
Wholegrain bread and products	−0.273	0.342	0.374
Wine	−0.109	0.370	0.041
Yogurt (full fat)	0.279	−0.218	0.354
Yogurt (low fat)	−0.174	0.496	0.098

* Foods with the highest factor loadings ≥0.3 and those between 0.25 to 0.3 are positively related to a dietary pattern. Foods with low factor loadings, and therefore less likely to be part of a dietary pattern, are between −0.25 and −0.3 and ≤−0.3.

**Table 3:** Results from multiple linear regression analysis evaluating the associations between lifestyle and socio-economic characteristics and dietary patterns extracted with principal component analysis in fasters (*n* = 200).

Dependent Variable	Variables	B	95% CI	P
**“Mixed Diet” pattern**	**Gender**	0.056	−0.49, 0.60	0.838
	**Education status**	−0.012	−0.23, 0.21	0.914
	**Family status**	−0.072	−0.41, 0.26	0.672
	**Smoking status**	−0.322	−0.70, 0.05	0.091
	**BMI status**	0.104	−0.25, 0.46	0.561
	**Body fat**	−0.006	−0.04, 0.03	0.748
	**PA status**	0.204	0.01, 0.40	0.039
	**Total energy (kcal)**	−0.001	0.00, 0.00	0.722
	**PRO (g)**	0.000	−0.02, 0.02	0.956
	**CHO (g)**	0.002	−0.01, 0.02	0.801
	**FAT (g)**	0.004	−0.03, 0.04	0.811
	**Waist circumference (cm)**	0.333	0.000, 0.66	0.050
	**HDL (mg/dL)**	−0.138	−0.50, 0.22	0.452
	**Triglycerides (mg/dL)**	−0.259	−0.64, 0.13	0.191
	**Glucose (mg/dL)**	−0.173	−0.77, 0.43	0.575
	**BP (mmHg)**	0.628	0.24, 1.01	0.002
	**MetS presence**	−0.375	−0.98, 0.23	0.224
**“Plant-based/Fasting Diet” pattern**	**Gender**	−0.123	−0.67, 0.42	0.660
	**Education status**	0.173	−0.05, 0.39	0.129
	**Family status**	0.200	−0.14, 0.54	0.248
	**Smoking status**	0.151	−0.22, 0.53	0.433
	**BMI status**	−0.013	−0.37, 0.34	0.945
	**Body fat**	0.000	−0.03, 0.03	0.992
	**PA status**	−0.050	−0.24, 0.14	0.619
	**Total energy (kcal)**	0.000	−0.004, 0.003	0.811
	**PRO (g)**	0.001	−0.01, 0.01	0.888
	**CHO (g)**	0.003	−0.01, 0.01	0.703
	**FAT (g)**	0.001	−0.03, 0.03	0.963
	**Waist circumference (cm)**	0.125	−0.22, 0.47	0.482
	**HDL (mmol/dL)**	−0.034	−0.41, 0.34	0.861
	**Triglycerides (mg/dL)**	0.219	−0.19, 0.63	0.295
	**Glucose (mg/dL)**	0.592	−0.04, 1.23	0.070
	**BP (mmHg)**	0.015	−0.39, 0.42	0.941
	**MetS presence**	−0.423	−1.06, 0.21	0.194

BMI: body mass index, PA: physical activity, HDL: high-density lipoprotein, BP: blood pressure, MetS: metabolic syndrome, PRO: protein, CHO: cholesterol.

**Table 4:** Results from multiple linear regression analysis evaluating the associations between lifestyle and socio-economic characteristics and dietary patterns extracted with principal component analysis in non-fasters (*n* = 200).

Dependent Variable	Variables	B	95% CI	P
**“Western Diet” pattern**	**Gender**	0.524	0.02, 1.02	0.039
	**Education status**	0.006	−0.21, 0.22	0.957
	**Family status**	−0.004	−0.28, 0.27	0.978
	**Smoking status**	0.009	−0.33, 0.35	0.957
	**BMI status**	0.126	−0.22, 0.47	0.477
	**Body fat**	−0.025	−0.06, 0.01	0.196
	**PA status**	0.046	−0.12, 0.22	0.607
	**Total energy (kcal)**	0.001	−0.001, 0.003	0.258
	**PRO (g)**	−0.006	−0.019, 0.007	0.353
	**CHO (g)**	−0.002	−0.010, 0.006	0.655
	**FAT (g)**	−0.013	−0.03, 0.006	0.172
	**Waist circumference (cm)**	−0.071	−0.43, 0.29	0.698
	**HDL (mmol/dL)**	0.379	−0.007, 0.76	0.054
	**Triglycerides (mg/dL)**	−0.005	−0.37, 0.36	0.978
	**Glucose (mg/dL)**	−0.240	−0.73, 0.25	0.335
	**BP (mmHg)**	0.157	−0.23, 0.54	0.430
	**MetS presence**	−0.345	−0.99, 0.30	0.296
**“Mixed Diet” pattern**	**Gender**	−0.142	−0.64, 0.35	0.576
	**Education status**	0.098	−0.11, 0.31	0.373
	**Family status**	−0.047	−0.32, 0.23	0.738
	**Smoking status**	−0.015	−0.35, 0.32	0.930
	**BMI status**	−0.256	−0.60, 0.09	0.151
	**Body fat**	0.029	−0.009, 0.06	0.132
	**PA status**	−0.109	−0.28, 0.06	0.224
	**Total energy (kcal)**	0.000	−0.002, 0.002	0.687
	**PRO (g)**	0.003	−0.01, 0.01	0.691
	**CHO (g)**	−0.001	−0.009, 0.007	0.771
	**FAT (g)**	0.000	−0.01, 0.01	0.985
	**Waist circumference (cm)**	−0.088	−0.45, 0.28	0.636
	**HDL (mmol/dL)**	−0.184	−0.57, 0.20	0.354
	**Triglycerides (mg/dL)**	−0.016	−0.38, 0.35	0.931
	**Glucose (mg/dL)**	−0.258	−0.75, 0.24	0.308
	**BP (mmHg)**	−0.084	−0.48, 0.31	0.679
	**MetS presence**	0.413	−0.24, 1.07	0.217
**“Mediterranean-like Diet” pattern**	**Gender**	0.511	0.03, 0.99	0.037
	**Education status**	−0.028	−0.23, 0.18	0.790
	**Family status**	0.438	0.17, 0.70	0.001
	**Smoking status**	−0.089	−0.41, 0.24	0.594
	**BMI status**	0.308	−0.02, 0.64	0.072
	**Body fat**	−0.017	−0.05, 0.01	0.346
	**PA status**	0.040	−0.12, 0.20	0.638
	**Total energy (kcal)**	0.000	−0.002, 0.001	0.603
	**PRO (g)**	0.004	−0.009, 0.01	0.527
	**CHO (g)**	0.002	−0.006, 0.01	0.563
	**FAT (g)**	0.004	−0.01, 0.02	0.679
	**Waist circumference (cm)**	0.415	0.05, 0.77	0.024
	**HDL (mmol/dL)**	−0.047	−0.43, 0.33	0.807
	**Triglycerides (mg/dL)**	−0.097	−0.46, 0.26	0.600
	**Glucose (mg/dL)**	0.233	−0.25, 0.72	0.347
	**BP (mmHg)**	0.294	−0.09, 0.68	0.138
	**MetS presence**	−0.342	−0.98, 0.30	0.296

BMI: body mass index, PA: physical activity, HDL: high-density lipoprotein, BP: blood pressure, MetS: metabolic syndrome, PRO: protein, CHO: cholesterol.

## Data Availability

The raw data supporting the conclusions of this study are available from the corresponding author upon request.

## Supplementary Materials

The following supporting information can be downloaded at: https://www.mdpi.com/article/10.3390/foods12183488/s1, Table S1. Demographic & lifestyle characteristics, and prevalence of metabolic syndrome and its components. Table S2. Details of the food items included in each of the 61 food groups for the principal component analysis. Table S3. Food groups and respective factor loadings in the dietary patterns of all study participants (n = 400). Table S4. Food groups and respective factor loadings in the dietary patterns in men (n = 143). Table S5. Food groups and respective factor loadings in the dietary patterns in women (n = 257). Table S6. Questionnaire used in the cross-sectional study in Greek language.
