# Supplementary material for: Association between Dietary Patterns and Metabolic Syndrome Risk Factors: A Cross-Sectional Study of Christian Orthodox Church Fasters and Non-Fasters in Greece
Source: Foods. 2023 Sep 19;12(18):3488. doi: 10.3390/foods12183488 (PMC10527694; doi:10.3390/foods12183488)
Supplement: Supplementary file 1 [file foods-12-03488-s001.zip › foods-2622855-supplementary.pdf]

Supplementary Tables

Table S1. Demographic & lifestyle characteristics, and prevalence of metabolic syndrome and its components.

Variable	Fasters (n=200)		Non-fasters (n=200)		p-value
	n	%	n	%	
**Sex**					0.60
Male	69	34.5	74	37.0	
Female	131	65.5	126	63.0	
**Education level**					0.17
Primary education	9	4.5	17	8.5	
Secondary education	51	25.5	54	27.0	
Tertiary education	113	56.5	104	52.0	
Master's/Doctoral	27	13.5	25	12.5	
**Marital status**					0.67
Single	88	44.0	94	47.0	
Married/ Living together	108	54.0	96	48.0	
Divorced	4	2.0	5	2.5	
Widowed	-		5	2.5	
**Smoking status**					0.000
Yes	11	5.5	58	71.0	
No – never	183	91.5	142	29.0	
No – quit smoking	6	3.0	-		
**Body Mass Index status**					0.15
Underweight (<18.5 kg/m^2)	-		2	1.0	
Normal weight (18.5-24.9 kg/m^2)	79	39.5	91	45.5	
Overweight (25-29.9 kg/m^2)	76	38.0	68	34.0	
Obesity (≥30 kg/m^2)	45	22.5	39	19.5	
**Supplement status**					0.71
Yes	43	21.5	40	20.0	
No	157	78.5	160	80.0	
**Way of eating**					0.004
Anxious	7	3.5	4	2.0	
Slowly	77	38.5	98	49.0	
Quickly	75	37.5	85	42.5	
Regular	41	20.5	13	6.5	
**Way of eating - company**					0.11
Sitting alone	48	24.0	47	23.5	
Sitting with someone	145	42.5	130	65.0	
In front of a TV/computer	7	3.5	23	11.5	
**Way of cooking**					0.47
Boiled	51	25.5	46	23.0	
Fried	117	58.5	122	61.0	
Grilled/oven	23	11.5	29	14.5	

Mixed methods	4	2.0	3	1.5	
**Use of salt apart of cooking process**					0.17
Yes	58	29.0	46	23.0	
No	142	71.0	154	77.0	
**Use of diet products**					0.002
Yes	36	18.0	62	31.0	
No	164	82.0	138	69.0	
**Physical activity level/status**					0.06
Extremely low *(never/rarely)*	12	6.0	27	13.5	
Low *(<2 times per week)*	57	28.5	53	26.5	
Moderate *(2-3 times per week)*	98	49.0	94	47.0	
High *(3-5 times per week)*	30	15.0	22	11.0	
Extremely high *(everyday)*	3	1.5	4	2.0	
**Free time workout**					0.26
Yes	74	37.0	85	42.5	
No	126	63.0	115	57.5	
	**Mean ± SD**		**Mean ± SD**		**p-value**
**Age (years)**	43.4 ± 16.7		41.9 ± 17.3		0.40
**WC >102cm for men or >88cm for women**	81 (40.5)		68 (34)		0.18
**FBG ≥100mg/dL**	13 (6.5)		28 (14.0)		0.013
**HDL cholesterol <40mg/dL for men or <50mg/dL for women**	62 (31.0)		56 (28)		0.51
**TRG ≥150mg/dL**	63 (31.5)		71 (35.5)		0.39
**BP ≥130/85mmHg**	34 (17.0)		67 (33.5)		<0.001
**MetS prevalence**	31 (15.5)		42 (21.0)		0.15

SD: Standard Deviation, WC: Waist Circumference, FBG: Fasting Blood Glucose, HDL: High Density Lipoprotein, TRG: Triglycerides, BP: Blood Pressure, MetS: Metabolic Syndrome.

Table S2. Details of the food items included in each of the 61 food groups for the principal component analysis.

a/a	Food groups	Original food(s) from the FFQ
1	Red meat	Pork
		Beef
		Lamb and goat
2	White meat	Chicken
		Rabbit
		Turkey
3	Processed meat	Sausage pork
		Sausage turkey
4	Fish fresh	Fish fresh

5	Fish canned	Fish canned
		Fish salty
6	Seafood	Seafood
		Shellfish
		Snail
7	Eggs	Egg
8	Soya products	Soya
		Cheese products from soya
		Milk products from soya
9	Legumes	Legumes
10	Nuts	Nuts
11	Pastitsio and mousaka	Pastitsio - mousaka
12	Pies homemade	Pies homemade
13	Pizza	Pizza
14	Vegetables homemade dishes	Stuffed peppers grape leaves cabbage with rice and meat
		Stuffed peppers grape leaves cabbage spinach with rice
		Vegetables cooked, ladera
15	Cheese full fat	Cheese wholefat, feta, kasseri, kefalotiri
		Cheese wholefat, graviera, parmezana
16	Cheese low fat	Cheese 12% fat, mizithra, anthotiro
		Cheese lowfat, milner, fina
		Cheese lowfat, cottage
17	Milk full fat	Milk goat
		Milk fresh full fat 30.5%
		Milk condensed full fat
		Milk choco beverage wholemilk
18	Milk low fat	Milk fresh low fat 10.5%
		Kefir & ariani
		Milk fresh nonfat
		Milk chocolate beverage nonfat
19	Yogurt full fat	Yogurt goat
		Yogurt cow full fat 10%
		Yogurt dessert
20	Yogurt low fat	Yogurt cow low fat 2%
		Yogurt cow nonfat
21	White bread and products	Bread white
		Breadsticks
		Bagel, koulouri
22	Whole meal bread and products	Bread wholegrain
		Breadsticks with seeds
23	Rusks	Rusk traditional
		Nut barley

		Nut wheat
		Rusk whole meal
		Rusk wheat
24	Salty bakery products	Bagel koulouri stuffed
25	Other bread	Pita bread Cypriot
		Pita bread
		Pita bread round
26	Pasta, rice, and other white cereals	Pasta white
		Rice white
		Traxana, couscous
27	Pasta, rice, and other whole meal cereals	Pasta whole meal
		Rice whole meal
28	Potatoes boiled	Potato boiled
29	Potatoes fried and roasted	Potato fried
		Potato roasted
30	Wholegrain breakfast cereals and muesli	Corn flakes whole meal
		Muesli oats
31	Other breakfast cereals	Corn flakes white
		Coco pops
		Corn flakes with chocolate
32	Cereal bar	Bars
33	Tahini and peanut butter spreads	Tahini
		Peanut butter
34	Sugar, honey and marmalade	Sugar
		Honey
		Marmalade
35	Vegetables raw	Vegetable fresh
		Corn
36	Vegetables not raw	Vegetable boiled
		Vegetable pickled
37	Fruits	Fruit fresh
		Fruit canned
		Fruit dried
38	Fruit juices	Fruit juice fresh
		Fruit juice canned
39	Soft drinks with sugar	Lemonade/orangeade with sugar
		Fizzy drink cola with sugar
		Fizzy drink with sugar other
40	Soft drinks without sugar	Fizzy drink cola light zero
		Fizzy drink light other
41	Energy drinks	Energy drinks
		Isotonic drinks

42	Butter	Butter
43	Other fat spreads	Margarine
		Becel
44	Olive oil and olives	Olive oil
		Olive
45	Other oils	Oilseed, sporelaio
46	Salad dressings and dips	Mayonnaise
		Mayonnaise light
		Ketchup
		Mustard
		Tarama
		Salad dip, tirokauteri, melitzanosalata
47	Coffee	Coffee nescafe, filtered, espresso
		Coffee greek
48	Cacao and chocolate powder	Cacao powder
		Chocolate powder or beverage
49	Tea	Tea green
		Tea black
		Tea herbal traditional
50	Wine	Wine
51	Beer	Beer
52	Ouzo, raki and tsipouro	Ouzo, raki, tsipouro
53	Liquor	Spirit beverages
54	Water	Water
55	Savoury snacks	Chips potato
56	Puddings	Pudding milk or rice full fat
57	Traditional confectionery	Donut loukoumas
		Dessert homemade, dessert spoon (koutaliou)
58	Chocolate confectionery	Dessert with chocolate
		Chocolates
59	Biscuits & cakes	Biscuits cake plain
		Biscuits cake with chocolate
60	Nougat & halvah	Nougat
		Nougat with nuts
		Halvah tahini
		Halvah semolina
61	Ice cream	Ice cream

Table S3. Food groups and respective factor loadings in the dietary patterns of all study participants (n=400).

Food group(s) *	Western Diet pattern	Mediterranean-like Diet pattern	Plant-based/Fasting Diet pattern
	Variance explained 7.2%	Variance explained 5.6%	Variance explained 4.7%
Beer	0.071	0.419	-0.107
Biscuits and cakes	0.390	-0.071	0.054
Butter	0.249	0.104	0.173
Cacao and chocolate powder	0.378	0.152	0.132
Cereal bar	0.170	0.409	0.121
Cheese full fat	0.163	-0.110	0.220
Cheese low fat	0.009	0.373	0.149
Chocolate confectionery	0.260	-0.012	-0.022
Coffee	-0.247	0.301	0.133
Eggs	0.041	-0.013	0.179
Energy drinks	0.257	0.192	-0.067
Fish canned	0.056	0.155	0.199
Fresh fish	0.017	0.007	0.220
Fruit juices	0.443	0.134	-0.016
Fruits	-0.131	-0.016	0.627
Ice-cream	0.404	-0.028	0.070
Legumes	0.246	-0.055	0.103
Liquor	0.043	0.493	-0.099
Milk full fat	0.505	-0.099	0.074
Milk low fat	-0.004	0.566	-0.043
Nougat halva	0.233	0.052	0.443
Nuts	-0.011	0.096	0.435
Olive oil olives	-0.257	0.096	0.450
Other bread	0.498	0.279	0.062
Other breakfast cereals	0.278	0.419	-0.050
Other fat spreads	0.128	-0.041	-0.068
Other oils	0.370	-0.205	0.128
Other seafood	0.162	0.052	0.337
Pasta rice and other white cereals	0.293	0.004	0.173
Pasta rice and other wholegrain cereals	0.148	0.253	0.102
Pastitsio	0.415	0.036	0.018
Pies	0.045	-0.051	0.082
Pizza	0.385	0.152	-0.009
Potatoes boiled	0.087	0.193	0.030
Potatoes other	0.351	0.043	-0.122

Processed meat	0.317	0.244	-0.312
Puddings	0.466	0.248	-0.045
Red meat	0.121	0.161	-0.106
Rusks	-0.018	0.318	0.243
Salad dressings dips	0.570	0.240	-0.069
Salty bakery products	0.306	0.216	-0.031
Savoury snacks	0.403	0.201	-0.104
Soft drinks with sugar	0.457	0.144	-0.276
Soft drinks without sugar	0.226	0.414	-0.170
Soya products	0.190	-0.154	0.130
Sugar honey marmalade	0.293	-0.064	0.102
Tahini and spreads	-0.006	-0.137	0.393
Tea	0.176	0.093	0.293
Traditional confectionery	0.381	-0.065	0.287
Traditional spirits	-0.107	0.396	0.068
Vegetables homemade dishes	0.142	0.081	0.324
Vegetables not raw	-0.182	-0.034	0.414
Vegetables raw	-0.062	0.215	0.303
Water	-0.046	0.034	0.151
White bread and products	0.271	-0.002	0.120
White meat	0.231	0.214	-0.131
Wholegrain breakfast cereals	0.101	0.480	0.256
Wholegrain bread and products	-0.314	0.368	0.336
Wine	-0.083	0.432	-0.044
Yogurt full fat	0.303	-0.152	0.321
Yogurt low fat	-0.225	0.463	0.138

**Foods that have the highest factor loadings ≥0.3 and those between 0.25 to 0.3, are positive related to the dietary pattern. Foods with low factor loadings for the dietary pattern, and therefore less likely to be part of that pattern, are between -0.25 and -0.3 and ≤ -0.3.*

Table S4. Food groups and respective factor loadings in the dietary patterns in men (n=143).

**Food group(s) ***	**Western Diet pattern**	**Mediterranean-like Diet pattern**
	Variance explained 8.7%	Variance explained 6.4%
Beer	0.342	0.106
Biscuits and cakes	0.416	-0.106
Butter	0.529	-0.022
Cacao and chocolate powder	0.460	0.204
Cereal bar	0.109	0.384
Cheese full fat	0.218	0.069

Cheese low fat	0.167	0.324
Chocolate confectionery	0.228	-0.132
Coffee	-0.108	0.287
Eggs	-0.047	0.130
Energy drinks	0.323	-0.029
Fish canned	0.257	0.194
Fresh fish	0.149	0.340
Fruit juices	0.448	0.087
Fruits	-0.072	0.681
Ice-cream	0.216	0.047
Legumes	-0.028	0.080
Liquor	0.333	0.137
Milk full fat	0.470	-0.137
Milk low fat	0.142	0.372
Nougat halva	0.254	0.285
Nuts	-0.012	0.440
Olive oil olives	0.047	0.497
Other bread	0.606	-0.054
Other breakfast cereals	0.312	0.140
Other fat spreads	0.186	-0.285
Other oils	0.385	-0.208
Other seafood	0.224	0.249
Pasta rice and other white cereals	0.315	0.055
Pasta rice and other whole meal cereals	0.299	0.275
Pastitsio	0.321	-0.103
Pies	0.147	-0.005
Pizza	0.298	0.058
Potatoes boiled	0.224	0.182
Potatoes other	0.320	-0.259
Processed meat	0.363	-0.247
Puddings	0.459	-0.227
Red meat	0.154	-0.032
Rusks	0.001	0.488
Salad dressings dips	0.472	-0.064
Salty bakery products	0.483	0.074
Savoury snacks	0.515	0.072
Soft drinks with sugar	0.616	-0.210
Soft drinks without sugar	0.465	0.071
Soya products	0.130	0.061
Sugar honey marmalade	0.223	-0.060
Tahini and spreads	-0.110	0.102
Tea	0.184	0.185

Traditional confectionery	0.342	0.167
Traditional spirits	0.139	0.179
Vegetables homemade dishes	0.161	0.289
Vegetables not raw	-0.130	0.307
Vegetables raw	-0.068	0.432
Water	-0.031	0.237
White bread and products	0.359	0.051
White meat	0.092	-0.065
Wholegrain breakfast cereals	0.278	0.386
Wholegrain bread and products	0.010	0.616
Wine	0.040	0.220
Yogurt full fat	0.278	0.104
Yogurt low fat	-0.043	0.489

**Foods that have the highest factor loadings ≥0.3 and those between 0.25 to 0.3, are positive related to the dietary pattern. Foods with low factor loadings for the dietary pattern, and therefore less likely to be part of that pattern, are between -0.25 and -0.3 and ≤ -0.3.*

Table S5. Food groups and respective factor loadings in the dietary patterns in women (n=257).

**Food group(s) ***	**Western Diet pattern**	**Mediterranean-like Diet pattern**
	Variance explained 7.5%	Variance explained 5.7%
Beer	-0.010	0.405
Biscuits and cakes	0.312	-0.104
Butter	0.142	0.085
Cacao and chocolate powder	0.322	0.050
Cereal bar	0.264	0.465
Cheese full fat	0.103	-0.129
Cheese low fat	0.034	0.401
Chocolate confectionery	0.247	-0.043
Coffee	-0.260	0.370
Eggs	0.065	-0.076
Energy drinks	0.235	0.168
Fish canned	-0.030	0.243
Fresh fish	-0.042	-0.126
Fruit juices	0.486	0.043
Fruits	-0.124	-0.121
Ice-cream	0.499	-0.064
Legumes	0.386	0.034
Liquor	-0.052	0.486
Milk full fat	0.456	-0.169

Milk low fat	0.088	0.624
Nougat halva	0.254	0.059
Nuts	0.038	0.036
Olive oil olives	-0.344	0.064
Other bread	0.476	0.216
Other breakfast cereals	0.293	0.409
Other fat spreads	0.050	0.005
Other oils	0.308	-0.225
Other seafood	0.155	0.015
Pasta rice and other white cereals	0.282	0.004
Pasta rice and other whole meal cereals	0.154	0.138
Pastitsio	0.454	0.025
Pies	-0.066	-0.084
Pizza	0.422	0.078
Potatoes boiled	0.051	0.165
Potatoes other	0.365	0.049
Processed meat	0.349	0.278
Puddings	0.465	0.299
Red meat	0.162	0.190
Rusks	0.116	0.322
Salad dressings dips	0.644	0.248
Salty bakery products	0.307	0.165
Savoury snacks	0.402	0.122
Soft drinks with sugar	0.388	0.055
Soft drinks without sugar	0.153	0.352
Soya products	0.207	-0.175
Sugar honey marmalade	0.250	-0.041
Tahini and spreads	-0.074	-0.145
Tea	0.139	0.064
Traditional confectionery	0.361	-0.039
Traditional spirits	-0.182	0.416
Vegetables homemade dishes	0.169	-0.007
Vegetables not raw	-0.237	-0.064
Vegetables raw	-0.015	0.203
Water	-0.033	-0.006
White bread and products	0.214	-0.035
White meat	0.298	0.237
Wholegrain breakfast cereals	0.069	0.448
Wholegrain bread and products	-0.350	0.332
Wine	-0.084	0.430
Yogurt full fat	0.266	-0.156
Yogurt low fat	-0.175	0.502

**Foods that have the highest factor loadings ≥0.3 and those between 0.25 to 0.3, are positive related to the dietary pattern. Foods with low factor loadings for the dietary pattern, and therefore less likely to be part of that pattern, are between -0.25 and -0.3 and ≤ -0.3.*

Table S6. Questionnaire used in the cross-sectional study in Greek language.

**Code**	Κωδικός αριθμός	
**Date**	Ημερομηνία επανελέγχου (Μήνας/ΗΗ/ΕΕΕΕ)	
**ΔΗΜΟΓΡΑΦΙΚΑ ΣΤΟΙΧΕΙΑ**		
**Age**	Ποια είναι η ηλικία σας;	...
**Sex**	Φύλο	1| Α<br>2| Γ
**Occup**	Ποιο το επάγγελμα που ασκείτε τώρα (τελευταίο έτος);	...
**Marrital**	Οικογενειακή κατάσταση	1|Άγαμος<br>2| Έγγαμος/Συζεί<br>3| Διαζευγμένος<br>4| Χήρος
**EDUC**	Σπουδές	1| Δημοτικό<br>2| Γυμνάσιο<br>3| Λύκειο<br>4| Πτυχιούχοι ΑΕΙ<br>5| Μεταπτυχιακές Σπουδές
**ΧΑΡΑΚΤΗΡΙΣΤΙΚΑ ΤΡΟΠΟΥ ΖΩΗΣ**		
**Smoking**	Είστε καπνιστής (περισσότερο από1 τσιγάρα ημερησίως το τελευταίο έτος);	1| ΝΑΙ<br>2| ΟΧΙ – Διέκοψα<br>0| ΟΧΙ – Δεν έχω καπνίσει ποτέ
**Sleep**	Πόσες ώρες ύπνου συμπληρώνετε την ημέρα;	

Κλίμακα IPAQ, Πηγή: Papathanasiou G, Georgoudis G, Papandreou M, Spyropoulos P, Georgakopoulos D, Kalfakakou V, Evangelou A. Reliability measures of the short International Physical Activity Questionnaire (IPAQ) in Greek young adults. Hellenic J Cardiol. 2009;50:283-94|		
**IPAQ_1**	Κατά τη διάρκεια των τελευταίων 7 ημερών, πόσες μέρες κάνατε έντονη σωματική δραστηριότητα όπως σκάψιμο, έντονη άσκηση με βάρη, τρέξιμο σε διάδρομο με κλίση, αεροβική γυμναστική, γρήγορο τρέξιμο, γρήγορη ποδηλασία, γρήγορη κολύμβηση, τένις, αγώνας σε γήπεδο (ποδόσφαιρο, basketball, volleyball, handball);	
**IPAQ_2**	Πόσο χρόνο (σε λεπτά) καταναλώσατε συνήθως κάνοντας έντονη σωματική δραστηριότητα σε μία από αυτές τις ημέρες;	
Πριν απαντήσετε στις ερωτήσεις 3 και 4 σκεφτείτε τις μέτριας έντασης δραστηριότητες που κάνατε τις τελευταίες 7 ημέρες. Μια μέτρια έντασης δραστηριότητα αναφέρεται σε δραστηριότητες που απαιτούν μέτρια σωματική δραστηριότητα και σας κάνουν να αναπνέετε κάπως δυσκολότερα από ότι συνήθως. Σκεφτείτε μόνο τις δραστηριότητες που κάνατε και είχαν διάρκεια τουλάχιστον 10 λεπτά.		
**IPAQ_3**	Κατά τη διάρκεια των τελευταίων 7 ημερών, πόσες μέρες κάνατε κάποια μέτρια σωματική δραστηριότητα όπως το να σηκώνετε και να μεταφέρετε ελαφρά βάρη (κάτω από 10 κιλά), συνολική καθαριότητα του σπιτιού, ήπιες ρυθμικές ασκήσεις σώματος, ποδηλασία αναψυχής με χαμηλή ταχύτητα, χαλαρή κολύμβηση σε μέτρια ένταση. Μη συμπεριλάβετε το περπάτημα. Να αναφερθούν μόνο οι δραστηριότητες που κάνατε για τουλάχιστον 10 λεπτά.	
**IPAQ_4**	Πόσο χρόνο (σε λεπτά) καταναλώσατε συνήθως κάνοντας σωματική δραστηριότητα μέτριας έντασης σε μία από αυτές τις ημέρες;	
**IPAQ_5**	Κατά τη διάρκεια των τελευταίων 7 ημερών, πόσες ημέρες περπατήσατε για τουλάχιστον10 λεπτά τη φορά;	
**IPAQ_6**	Πόσο χρόνο (σε λεπτά) καταναλώσατε συνήθως για περπάτημα σε μια από τις παραπάνω ημέρες;	
**IPAQ_7**	Κατά τη διάρκεια των τελευταίων 7 ημερών, πόσο χρόνο (σε ώρες συνολικά) καταναλώσατε καθιστός στο σπίτι, στο γραφείο, με φίλους, ή διαβάζοντας, ξαπλώνοντας ή παρακολουθώντας τηλεόραση; Μην συμπεριλάβετε τις ώρες ύπνου.	

**ΔΙΑΤΡΟΦΙΚΗ ΑΞΙΟΛΟΓΗΣΗ**	
**24hr**	Παρακαλώ συμβουλευτείτε τον ερευνητή για να συμπληρώσετε μαζί την 24ώρη ανάκλησή σας.
	Κενός χώρος για καταγραφή 24ώρης ανάκλησης
ΕΡΩΤΗΜΑΤΟΛΟΓΙΟ ΣΥΧΝΟΤΗΤΑΣ ΚΑΤΑΝΑΛΩΣΗΣ ΤΡΟΦΙΜΩΝ<br>***Πηγή***: Bountziouka V, Bathrellou E, Giotopoulou A, Katsagoni C, Bonou M, Vallianou N, Barbetseas J, Avgerinos PC, Panagiotakos DB. Development, repeatability and validity regarding energy and macronutrient intake of a semi-quantitative food frequency questionnaire: methodological considerations. Nutr Metab Cardiovasc Dis. 2012;22:659-67.	

**Σημειώστε ΠΟΣΟ ΣΥΧΝΑ καταναλώσατε τα παρακάτω τρόφιμα τον <u>τελευταίο μήνα.</u>** Προσοχή, θα πρέπει να απαντήσετε έχοντας ως **<u>μερίδα αναφοράς την ποσότητα που αναγράφεται</u>** στις παρενθέσεις. (Συντμήσεις: *φ* = φορές, *γρ.* = γραμμάρια, *τμχ.* = τεμάχιο, *φλ.* = φλιτζάνι τσαγιού = 240 ml)	**1| Ποτέ/ Σπάνια**	**2| 1-3 φ/ μήνα**	**3| 1-2 φ/ εβδομ.**	**4| 3-6 φ/ εβδομ.**	**5| 1 φ/ ημέρα**	6| **≥ 2 φ/ ημέρα**
DQ_1|Γάλα/ γιαούρτι πλήρες (1 ποτήρι/ 1 κεσεδάκι)	1	2	3	4	5	6
DQ_2|Γάλα/ γιαούρτι χαμηλό σε λιπαρά (1 ποτήρι/ 1 κεσεδάκι)	1	2	3	4	5	6
DQ_3|Τυρί κίτρινο, τυρί σε κρέμα (30 γρ)	1	2	3	4	5	6
DQ_4|Τυρί φέτα, ανθότυρο (30 γρ)	1	2	3	4	5	6
DQ_5|Τυρί άπαχο ή χαμηλό σε λιπαρά (light, κότατζ) (30 γρ)	1	2	3	4	5	6
DQ_6|Αυγό (βραστό, τηγανιτό, ομελέτα) (1 τμχ)	1	2	3	4	5	6
DQ_7|Ψωμί άσπρο (1 φέτα 30γρ ή φέτα τοστ), φρυγανιά (2 τμχ)	1	2	3	4	5	6
DQ_8|Ψωμί ολικής αλέσεως (1 φέτα 30γρ ή φέτα τοστ), φρυγανιά (2 τμχ)	1	2	3	4	5	6
DQ_9|Κουλούρι Θεσ/κης, πίτα (σουβλάκι), ψωμάκια μπέργκερ (1 τμχ)	1	2	3	4	5	6
DQ_10|Κριτσίνια (2 λεπτά), παξιμάδια (1 μέτριο), κουλούρια (2 μέτρια)	1	2	3	4	5	6
DQ_11|Δημητριακά πρωινού (½ φλ), μπάρες δημητριακών (1 τμχ)	1	2	3	4	5	6
DQ_12|Ρύζι λευκό (1 φλ)	1	2	3	4	5	6
DQ_13|Ρύζι καστανό (1 φλ)	1	2	3	4	5	6
DQ_14|Μακαρόνια, κριθαράκι, χυλοπίτες, άλλα ζυμαρικά (1 φλ)	1	2	3	4	5	6
DQ_15|Ζυμαρικά ολικής αλέσεως (1 φλ)	1	2	3	4	5	6
DQ_16|Πατάτες βραστές, φούρνου, πουρές (1 μέτρια/ ½ φλ)	1	2	3	4	5	6

DQ_17|Πατάτες τηγανιτές (½ μερίδα εστιατορίου)	1	2	3	4	5	6
DQ_18|Μοσχάρι (μπριζόλα, κομμάτι) (150 γρ)	1	2	3	4	5	6
DQ_19|Μπιφτέκι (2 τμχ), κεφτεδάκια (4 τμχ), κιμάς (1 κουτάλα)	1	2	3	4	5	6
DQ_20|Κοτόπουλο/ γαλοπούλα (όλα τα είδη) (150 γρ)	1	2	3	4	5	6
DQ_21|Χοιρινό (μπριζόλα, κομμάτι, σουβλάκι) (150 γρ)	1	2	3	4	5	6
DQ_22|Πίτσα (1 κομμάτι,)	1	2	3	4	5	6
DQ_23|Γύρος (100 γρ)	1	2	3	4	5	6
DQ_24|Αρνί, κατσίκι, κυνήγι, παϊδάκια (150 γρ)	1	2	3	4	5	6
DQ_25|Αλλαντικά (1 φέτα)	1	2	3	4	5	6
DQ_26|Λουκάνικα (1 μέτριο), μπέικον (2 φέτες)	1	2	3	4	5	6
DQ_27|Αλλαντικά/ κρεατοσκευάσματα άπαχα ή light (όπως παραπάνω)	1	2	3	4	5	6
DQ_28|Ψάρια μικρά (150 γρ)	1	2	3	4	5	6
DQ_29|Ψάρια μεγάλα (150 γρ)	1	2	3	4	5	6
DQ_30|Θαλασσινά (χταπόδι, καλαμάρι, γαρίδες) (150 γρ)	1	2	3	4	5	6
DQ_31|Όσπρια (π.χ. φακές, φασόλια, ρεβίθια) (1 πιάτο)	1	2	3	4	5	6
DQ_32|Σπανακόρυζο/ λαχανόρυζο (1 πιάτο), γεμιστά (2 μέτρια)	1	2	3	4	5	6
DQ_33|Παστίτσιο, μουσακάς, παπουτσάκια (1 μερίδα = 150 γρ)	1	2	3	4	5	6
DQ_34|Αρακάς, φασολάκια, μπάμιες, αγκινάρες (1 πιάτο)	1	2	3	4	5	6
DQ_35|Τομάτα, αγγούρι, καρότο, πιπεριά (1 φλ. ωμά)	1	2	3	4	5	6

DQ_36|Μαρούλι, λάχανο, σπανάκι, ρόκα (1 φλ. ωμά)	1	2	3	4	5	6
DQ_37|Μπρόκολο, κουνουπίδι, κολοκυθάκια, (½ φλ. βραστά)	1	2	3	4	5	6
DQ_38|Χόρτα, πράσο, σπανάκι, σέλινο (½ φλ. βραστά)	1	2	3	4	5	6
DQ_39|Πορτοκάλι (1 μέτριο)	1	2	3	4	5	6
DQ_40|Μήλο, αχλάδι (1 μέτριο)	1	2	3	4	5	6
DQ_41|Άλλα χειμερινά φρούτα (1 ολόκληρο ή ½ φλ)	1	2	3	4	5	6
DQ_42|Μπανάνα (1 μέτρια)	1	2	3	4	5	6
DQ_43|Άλλα καλοκαιρινά φρούτα (1 ολόκληρο ή ½ φλ)	1	2	3	4	5	6
DQ_44|Χυμός φρούτων (1 ποτήρι)	1	2	3	4	5	6
DQ_45|Αποξηραμένα φρούτα (¼ φλ.)	1	2	3	4	5	6
DQ_46|Ξηροί καρποί, σπόροι (1 φλιτζανάκι καφέ)	1	2	3	4	5	6
DQ_47|Πίτες σπιτικές (π.χ. τυρόπιτα, σπανακόπιτα) (1 κομμάτι)	1	2	3	4	5	6
DQ_48|Πίτες έτοιμες (1 κομμάτι)	1	2	3	4	5	6
DQ_49|Τοστ, σάντουιτς (1 ολόκληρο)	1	2	3	4	5	6
DQ_50|Γλυκά ταψιού (1 τμχ)	1	2	3	4	5	6
DQ_51|Γλυκά κουταλιού, κομπόστα, ζελέ (1 μερίδα)	1	2	3	4	5	6
DQ_52|Πάστες, τάρτα (1 τμχ)	1	2	3	4	5	6
DQ_53|Κρουασάν (1), γκοφρέτες (1 μέτρια), κέικ (1 φέτα), μπισκότα (3-4)	1	2	3	4	5	6
DQ_54|Σοκολάτα (όλα τα είδη) (1 μέτρια ~ 60 γρ)	1	2	3	4	5	6
DQ_55|Παγωτό, μιλκ σέικ, κρέμα, ρυζόγαλο (1 τμχ)	1	2	3	4	5	6
DQ_56|Πατατάκια, γαριδάκια, ποπ κορν (1 σακουλάκι ~70 γρ)	1	2	3	4	5	6

DQ_57|Μέλι, μαρμελάδα, ζάχαρη (π.χ. σε ψωμί, καφέ) (1 κουτ. γλυκού)	1	2	3	4	5	6
DQ_58|Ελιές (10 μικρές/ 5 μεγάλες)	1	2	3	4	5	6
DQ_59|Μαγιονέζα, σως (1 κουτ. σούπας)	1	2	3	4	5	6
DQ_60|Μαγιονέζα/ σως λάιτ (1 κουτ. σούπας)	1	2	3	4	5	6
DQ_61|Πόσες φορές χρησιμοποιείτε ελαιόλαδο (οπουδήποτε);	1	2	3	4	5	6
DQ_62|Πόσες φορές χρησιμοποιείτε σπορέλαιο (οπουδήποτε);	1	2	3	4	5	6
DQ_63|Πόσες φορές χρησιμοποιείτε μαργαρίνη (οπουδήποτε);	1	2	3	4	5	6
DQ_64|Πόσες φορές χρησιμοποιείτε βούτυρο (οπουδήποτε);	1	2	3	4	5	6
DQ_65|Κρασί (1 ποτήρι = 125 ml)	1	2	3	4	5	6
DQ_66|Μπύρα (1 ποτήρι = 240 ml)	1	2	3	4	5	6
DQ_67|Άλλο είδος αλκοόλ (1 ποτό)	1	2	3	4	5	6
DQ_68|Αναψυκτικά (1 κουτί ~ 330 ml)	1	2	3	4	5	6
DQ_69|Αναψυκτικά light (1 κουτί ~ 330 ml)	1	2	3	4	5	6
DQ_70|Καφές, συνολικά (1 φλ. ή ποτήρι)	1	2	3	4	5	6
DQ_71|Τσάι, άλλα αφεψήματα (1 φλ)	1	2	3	4	5	6
DQ_72|Καφές ζεστός, φίλτρου ή τύπου καπουτσίνο (1 φλιτζάνι = 240 ml)	1	2	3	4	5	6
DQ_73|Καφές Ελληνικός ή εσπρέσο (1 μικρό φλιτζάνι = 40 ml)	1	2	3	4	5	6
DQ_74|Καφές κρύος, Φραπέ ή Φρέντο (1 ποτήρι = 240 ml)	1	2	3	4	5	6
DQ_75|Σοκολάτα ρόφημα, ζεστή ή κρύα (1 φλιτζάνι = 240 ml)	1	2	3	4	5	6

	ΑΠΟΤΙΜΗΣΗ ΔΙΑΤΡΟΦΙΚΩΝ ΣΥΜΠΕΡΙΦΟΡΩΝ				
	**Πόσες φορές την εβδομάδα καταναλώνετε;**				
ΝΗ_1	Πρωινό	**1|** σπάνια	**2|** 1-2 φορές/εβδ	**3|** 3-5 φορές/εβδ	**4|** σχεδόν κάθε μέρα
ΝΗ_3	Πρόγευμα	**1|** σπάνια	**2|** 1-2 φορές/εβδ	**3|** 3-5 φορές/εβδ	**4|** σχεδόν κάθε μέρα
ΝΗ_4	Μεσημεριανό	**1|** σπάνια	**2|** 1-2 φορές/εβδ	**3|** 3-5 φορές/εβδ	**4|** σχεδόν κάθε μέρα
ΝΗ_6	Απογευματινό	**1|** σπάνια	**2|** 1-2 φορές/εβδ	**3|** 3-5 φορές/εβδ	**4|** σχεδόν κάθε μέρα
ΝΗ_7	Βραδινό	**1|** σπάνια	**2|** 1-2 φορές/εβδ	**3|** 3-5 φορές/εβδ	**4|** σχεδόν κάθε μέρα
ΝΗ_9	Προ του ύπνου	**1|** σπάνια	**2|** 1-2 φορές/εβδ	**3|** 3-5 φορές/εβδ	**4|** σχεδόν κάθε μέρα
ΝΗ_5	*Κατανάλωση αλκοόλ με το Μεσημεριανό γεύμα;*	0|ΟΧΙ	1| κρασί κόκκινο	2| κρασί άσπρο	3| μπύρα; 4| άλλο
ΝΗ_8	*Κατανάλωση αλκοόλ με το Βραδινό γεύμα;*	0|ΟΧΙ	1| κρασί κόκκινο	2| κρασί άσπρο	3| μπύρα; 4| άλλο
ΝΗ_10	Πόση ώρα μετά το βραδινό φαγητό κοιμάστε; Βραδινό ή προ του ύπνου γεύμα (min)				
ΝΗ_11	Παραλείπετε κάποιο-α γεύματα λόγω φόρτου εργασίας;	**1|** < 1φορά/3μηνο	**2|** 1-3 φορές/μήνα	**3|** 2-4 φορές/εβδομάδα	**4|** σχεδόν κάθε μέρα
ΝΗ_12	Παραλείπετε κάποιο-α γεύματα γιατί θέλετε να χάσετε βάρος;	**1|** < 1φορά/3μηνο	**2|** 1-3 φορές/μήνα	**3|** 2-4 φορές/εβδομάδα	**4|** σχεδόν κάθε μέρα
ΝΗ_13	Παραλείπετε κάποιο-α γεύματα γιατί απλά εκείνη την ώρα δεν πεινάτε;	**1|** < 1φορά/3μηνο	**2|** 1-3 φορές/μήνα	**3|** 2-4 φορές/εβδομάδα	**4|** σχεδόν κάθε μέρα
ΝΗ_14	Κατανάλωση τροφής μπροστά στην τηλεόραση	**1|** < 1φορά/3μηνο	**2|** 1-3 φορές/μήνα	**3|** 2-4 φορές/εβδομάδα	**4|** σχεδόν κάθε μέρα
ΝΗ_15	Κατανάλωση τροφής κάτω από συνθήκες πίεσης (βιασύνη, άγχος)	**1|** σπάνια	**2|** 1-2 φορές την εβδομάδα	**3|** 3-5 φορές την εβδομάδα	**4|** σχεδόν κάθε μέρα
ΝΗ_16	Κατανάλωση τροφής ενώ ταυτόχρονα εργάζεστε (όχι σε διάλειμμα)	**1|** σπάνια	**2|** 1-2 φορές την εβδομάδα	**3|** 3-5 φορές την εβδομάδα	**4|** σχεδόν κάθε μέρα
ΝΗ_17	Κατανάλωση τροφής ενώ είστε όρθιος ή περπατάτε	**1|** σπάνια	**2|** 1-2 φορές την εβδομάδα	**3|** 3-5 φορές την εβδομάδα	**4|** σχεδόν κάθε μέρα
ΝΗ_18	Πόσο συχνά καταναλώνετε κάποιο πιο βαρύ γεύμα που προκαλεί δυσφορία;	**1|** < 1φορά/3μηνο	**2|** 1-4 φορές/μήνα	**3|** 2-4 φορές/εβδομάδα	**4|** σχεδόν κάθε μέρα

NH_19	Εσείς είστε υπεύθυνος-η για την προετοιμασία του φαγητού;	**1|** σπάνια	**2|** μερικές φορές την εβδομάδα	**3|** για κάποιο γεύμα της ημέρας	**4|** σχεδόν για όλα τα γεύματα
NH_20	Πόσο συχνά τρώτε μόνος-η;	**1|** σπάνια	**2|** μερικές φορές την εβδομάδα	**3|** κάποιο γεύμα της ημέρας	**4|** σχεδόν όλα τα γεύματα
